# The limits of scaling in aggregation-driven patterning of cell collectives

**DOI:** 10.1073/pnas.2617301123

**Published:** 2026-11-02

**Authors:** Michael L. Zhao, Jan Rombouts, Anniek Stokkermans, Anna Erzberger, Alexander Aulehla

**Affiliations:** ^a^Developmental Biology Unit, European Molecular Biology Laboratory, Heidelberg 69117, Germany; ^b^Cell Biology and Biophysics Unit, European Molecular Biology Laboratory, Heidelberg 69117, Germany; ^c^ Unit of Theoretical Chronobiology, Faculté des Sciences, Université libre de Bruxelles, Brussels 1050, Belgium; ^d^Hubrecht Institute, Utrecht 3584 CT, Netherlands

**Keywords:** scaling, embryonic cell collectives, self-organization, cell aggregation, patterning

## Abstract

Robust development requires maintaining correct proportions as overall size varies. What controls and limits this scaling remains poorly understood due to the complex interplay of mechanical and biochemical embryonic factors. Combining confinement of dissociated embryonic presomitic mesoderm cells with imaging and chemical perturbations, we identified cell aggregation as the initial event in de novo posterior pole formation. Using a continuum model based solely on cell–cell attraction, we quantitatively map how the time available for aggregation-driven patterning limits the maximum scalable system size. Small systems allow rapid, robust pattern scaling, whereas coarsening dynamics substantially delay scaled patterns in large systems. Our experiments quantitatively confirm these predicted scaling regimes. Together, our results reveal a developmental time–size tradeoff governing the scaling of aggregation-driven patterns.

## Significance

The ability to scale cell fate patterns with size is a striking hallmark of embryonic development, enabling embryos of different sizes to form correctly proportioned body plans. Here, we ask what limits this scaling capacity. Using a self-organizing embryonic cell system, we show that small systems form a single pole whose size scales with overall system size. In contrast, larger systems form multiple poles and no longer follow the same scaling relationship. Both outcomes arise from the same aggregation mechanism, but scaling ultimately fails in larger systems because the time required to reach a single-pole pattern exceeds developmental timescales. This work reveals a fundamental time–size tradeoff that sets the limits of scaling in aggregation-driven cell fate patterning.

How cell fate patterns change with the size of multicellular systems is a fundamental question. Developing embryos exhibit the remarkable ability to scale cell fate patterns with overall system size (1–5). Such scaling enables downstream morphogenetic processes to generate proportionately organized tissues despite variation in embryo size (6–8). This important property of living systems raises a series of interconnected questions. What mechanisms underlie cell fate patterning and determine its ability to scale? How are the size limits to pattern scaling established?

Considerable progress has been made in identifying mechanisms that enable patterns to scale with overall system size (9–11). Morphogen gradients are among the best-studied examples, notably the Bicoid gradient in *Drosophila* embryos (3). These studies have revealed diverse strategies that couple patterning to system size, including source–sink arrangements (12), size-dependent reaction or transport processes (1, 13), feedback regulation (14), and changes in cellular signaling responses (15). Related size-coupling mechanisms can also modify self-organized reaction–diffusion systems, allowing their intrinsic patterning length scales to adjust with system size (16).

Multicellular patterns can also emerge through cell movements, which play important roles across diverse biological contexts (17–25). Cell aggregation is one common form of movement-driven patterning, occurring across both unicellular and multicellular organisms. Examples include aggregative multicellularity in holozoans, chemotactic aggregation in *Dictyostelium*, neutrophil swarming, and precartilage condensation during chondrogenesis (26–29). Because movement-driven patterns depend on cells physically redistributing in space, changes in system size may alter both the distances over which cells must move and the number of cells whose movements must be coordinated, with potential consequences for the spacing and position of the final pattern. Compared with gradient-mediated patterns, it remains less understood how movement-driven patterns become coupled to system size. In particular, it is unclear whether this coupling requires a dedicated size-dependent regulatory mechanism, analogous to expander-like feedback in reaction–diffusion systems, or whether pattern scaling can arise without explicit size-dependent regulation.

Defining the size range over which scaling holds can help distinguish among scaling strategies and reveal the constraints that patterning systems face as tissue size changes. However, experimentally mapping both the scaling regime and its limit remains challenging. Studies of naturally varying and experimentally manipulated embryos have demonstrated robust scaling across embryo sizes that remain compatible with development (1, 2, 8). These studies reveal how scaling operates within that range, but provide more limited access to the broader size variation needed to determine where scaling breaks down. Tissue regeneration provides one route to study pattern scaling over larger size ranges than those observed during normal development (30). In vitro models derived from primary tissue or cultured embryonic stem cells provide a complementary approach by enabling experimental manipulation of size, density, and spatial confinement in ways that are difficult to achieve in native biological contexts (31–40). These systems therefore enable system size to be varied systematically while patterning outcomes are measured, allowing scaling and its limits to be tested in a controlled setting.

In this study, we adapted an ex vivo model of embryonic posterior pole formation (35, 41) to investigate how patterns change with system size. Imaging and chemical perturbation experiments identified cell aggregation as a key early process during pattern formation. We used this observation to formulate a continuum description of cell–cell attraction, which predicts specific time-dependent limits of pattern scaling. These findings quantitatively resolve how the finite time available for aggregation-driven patterning limits the system size over which pattern scaling can be achieved, revealing a tradeoff between the timescale of cell-attraction-based patterning and the spatial range over which scaling remains accessible.

## Results

### Limits to Scaling Are Observed in Ex Vivo Posterior Fate Patterns.

**Fig. 1.**
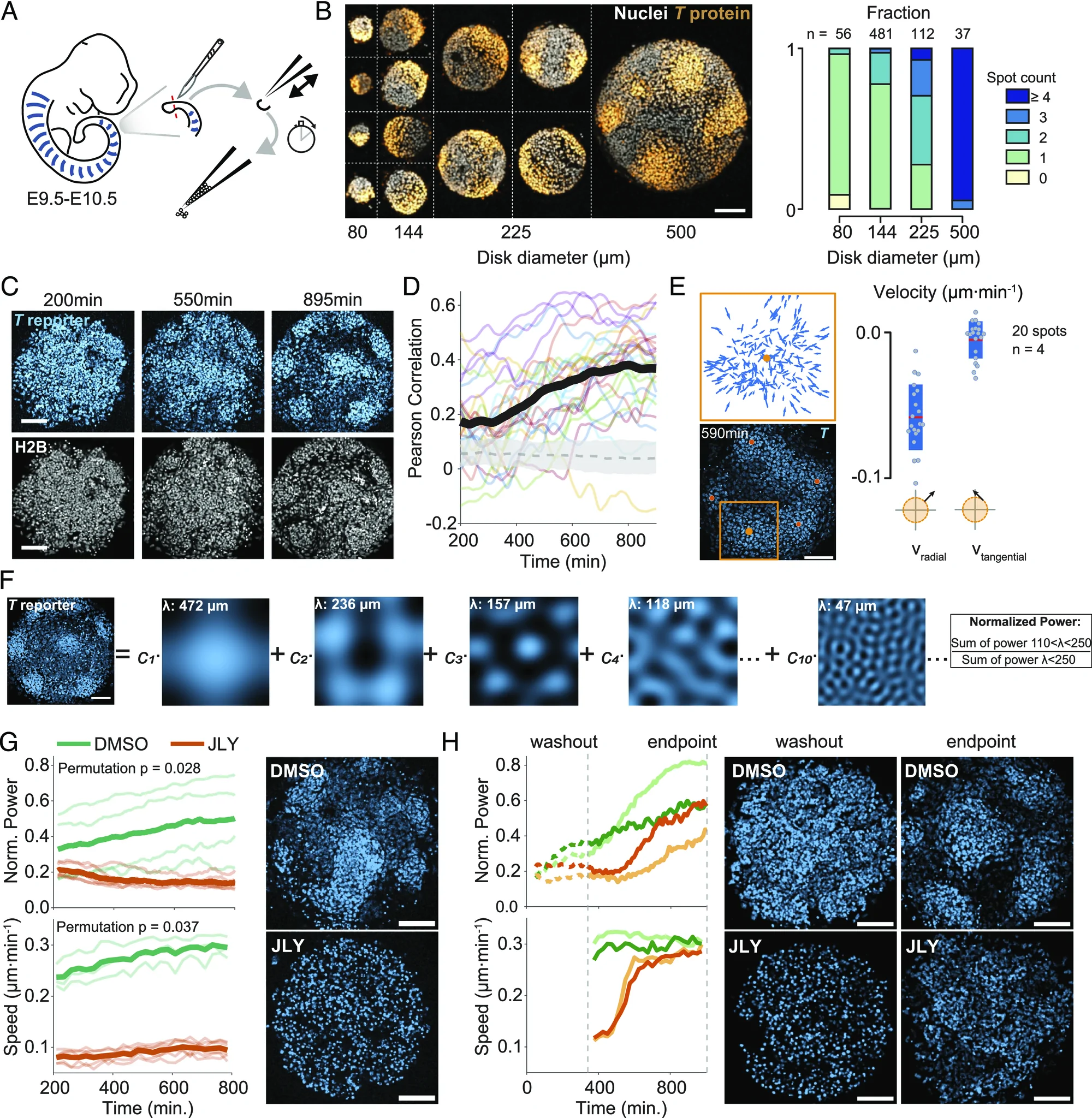
Limited scaling of *T* patterns in the ePSM assay. (*A*) We dissociate PSM tissue from mouse embryos as schematically shown, and define time 0 min in all subsequent analyses as the point of dissociation. (*B*) We visualized *T* protein levels by immunofluorescence staining (nuclei: DAPI, *T* protein: Alexa-Fluor 568) in PSM cells on disks of different diameters at t=1,140 min post dissociation (*Left*: example composite image formed by manual tiling, *Right*: quantification of *T* spot numbers). (Scale bar, 100 μm.) (*C*) Example montage shows the dynamic progression of *T* patterning after dissociation of PSM cells. (Scale bar, 100 μm.) (*D*) The Pearson correlation between local *T* activity and local cell density for 27 wells, represented by thin colored lines, increases over time. Mean of all wells is shown by the bold black line. Local areas were obtained by discretizing images onto a 50 μm × 50 μm square grid. Gray dashed line and band represent median and 95th percentile bands of a null relationship obtained by random rotations and reflection of the local *T* activity field relative to the local cell density field. (*E*) An example of quantified cell velocity vectors obtained from single cell tracking are shown in a 200 μm × 200 μm region (orange square) around one of five manually identified *T* spots (orange dots) from 200 to 590 min. The time-averaged radial velocities of the cells around *T* spot centers collected from four different wells (*Right*) are shown together with the mean (red line) and SD (blue box). (*F*) To quantify *T* patterning dynamics, we compute the normalized power of the *T* reporter signal, defined as shown schematically. (Scale bar, 100 μm.) (*G*) Effect of JLY treatment on the normalized power and cell speed compared to control (DMSO) samples (thin lines: individual well trajectories; bold lines: mean; *P*-values: permutation test of the treatment × time interaction for power and permutation test for difference in average starting speed). Cell speed and normalized power are calculated for the same samples with the exception of one experimental day for which matched cell-tracking data were unavailable. Images represent examples for each treatment condition. (Scale bar, 100 μm.) (*H*) Effect of JLY washout on the normalized power and cell speed. Images represent examples for each treatment condition before and after JLY washout. (Scale bar, 100 μm.)

Dissociated cells derived from mouse embryonic presomitic mesoderm (PSM) form de novo structures termed emergent PSMs (ePSMs) [Fig. 1*A*, 35, 41). ePSMs have been previously shown to recapitulate key features of posterior fate organization in the PSM, including expression of the posterior marker Brachyury (*T*) in a radial arrangement in which central positions correspond to more posterior identities and peripheral positions to more anterior identities (35). We exploited the approximately two-dimensional organization of ePSMs to establish an imaging workflow that captured both single-cell behavior and the mesoscale *T* expression pattern. Combined with micropatterning to control cell attachment area, this system provided a tractable setting in which to test how posterior fate patterns depend on system size.

To determine how patterning depends on system size, we seeded dissociated PSM cells on fibronectin-coated disks with diameters ranging from 80 μm to 500 μm. Cells were fixed after either 4 or 19 h and stained for *T* protein. Samples fixed after 4 h confirmed that dissociation generated approximately homogeneous spatial *T* profiles (*SI Appendix*, Fig. S1*A*). By 19 h, cells had formed discrete spot-like *T* patterns (Fig. 1*B*). The number of *T* spots increased with system size: for the larger disks (225 μm and 500 μm), the dominant outcome was a multispot pattern, whereas for the smaller disks (80 μm and 144 μm), the dominant outcome was a single *T* spot (Fig. 1*B* and *SI Appendix*, Fig. S1*B*). Restricting the analysis to single-spot patterns, we found that spot diameter and area increased with disk size (*SI Appendix*, Fig. S1 *B*–*D*). Thus, ePSMs exhibit a limited scaling regime in which a unipolar *T* pattern scales with system size, but above a critical size the dominant outcome shifts to multipolar patterns. The size at which this happens defines the boundary of the scaling regime. This size dependence on simple disk geometries resembles recent observations in other models of A–P patterning, suggesting that shared patterning principles may operate across these systems (42, 43). To understand the basis of this scaling limit, we next investigated the dynamical mechanisms underlying *T* pattern formation.

We performed time-lapse fluorescence imaging of dissociated PSM cells from a *T* reporter mouse line to capture the onset of pattern formation (Movie S1, 44). Western blotting and single-cell analysis confirmed that the reporter faithfully reflected *T* protein levels throughout the patterning time course (*SI Appendix*, Fig. S2 *A*–*C*). We imaged cells on fibronectin-coated disks of approximately 450 μm diameter, a system size well above the scaling limit and within the multispot regime. Following a 200-min period to allow for cell attachment and spreading, we observed dissociated cells forming spot-like *T* patterns that were accompanied by the emergence of dense cell clusters (Fig. 1*C* and *SI Appendix*, Fig. S3 *A* and *B*). The average nearest-neighbor distance between manually identified *T* spots was 164 μm and remained consistent across the wells observed, in agreement with previous ePSM measurements (*SI Appendix*, Fig. S3 *C* and *D*, 35). Over the course of pattern formation, local *T* reporter activity became positively correlated with local cell density (Fig. 1*D*). The appearance of dense clusters, together with this positive correlation, suggested that cell movements contribute to *T* pattern formation. To examine this possibility, we tracked cell nuclei during the first half of the experiment, between 200 and 590 min post dissociation. To determine how cells incorporated into developing *T* spots reached these regions, we identified cells located within 60 μm of manually identified spot centers at the end of the tracking interval and reconstructed their preceding movements. These cells showed a pronounced inward radial bias, consistent with cellular aggregation (Fig. 1*E*). Consistent with this interpretation, particle image velocimetry showed that cell flows across the well tended to converge on future sites of high *T* gene activity (*SI Appendix*, Fig. S4 *A*–*D*). To determine whether *T* levels at the beginning of the tracking interval were associated with aggregation propensity, we asked whether *T* reporter activity at 200 min post dissociation predicted subsequent aggregation-directed movement. Cells with above-median initial *T* reporter activity showed stronger inward radial movement toward future spot centers than cells with below-median reporter activity, whereas their tangential velocities did not differ (*SI Appendix*, Fig. S4 *E* and *F*). Directed, aggregation-like cell movements therefore accompany the initial formation of *T* patterns and are stronger among cells with higher initial *T* reporter activity.

To test whether cell movements are required for the initial formation of regions marked by *T*, we applied a pharmacological cocktail chosen to broadly suppress movement within the cell population. Specifically, we used the inhibitor combination Jasplakinolide, Latrunculin A, and Y-27632 (JLY), previously described to arrest actin dynamics (45). To quantify the effect on spatial patterning, we used Fourier analysis to decompose *T* reporter images into spatial modes, each associated with a characteristic wavelength (Fig. 1*F*). We then defined a normalized power score as the summed contribution of modes with wavelengths between 110 and 250 μm, consistent with the observed nearest-neighbor spacing, divided by the total power across all modes of 250 μm or less (Fig. 1*F*). JLY treatment strongly reduced both cell speed and pattern progression, as quantified by cell speed and normalized power, respectively (Fig. 1*G*). Washout after 6 h of JLY treatment showed that both effects were partially reversible following media replacement (Fig. 1*H* and Movies S2 and S3). Notably, cell speed began to recover before *T* patterning resumed, consistent with cell movement acting upstream of visible *T* pattern formation. These experiments provide evidence that aggregation-like cell movements are required for the initiation of *T* pattern formation.

### Cell Aggregation Precedes Changes in T Expression.

**Fig. 2.**
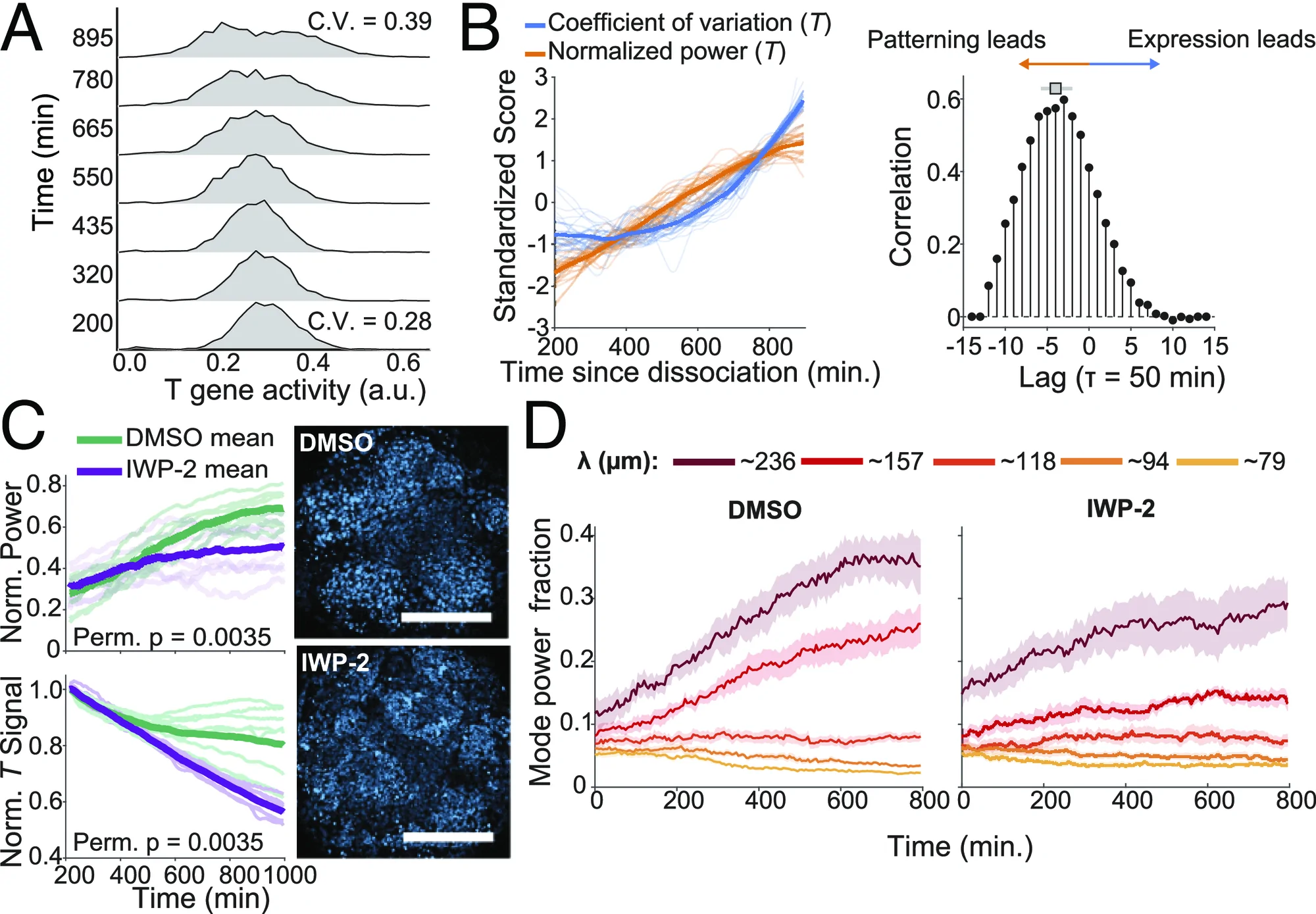
Cell aggregation precedes changes in *T* gene activity. (*A*) Time evolution of the *T* gene activity distribution across the population of cells in one well. (*B*) Comparison of standardized scores of the normalized power of cell nuclei patterns quantified from *T* reporter images (orange) to the *T* distribution coefficient of variation (blue) for 27 wells. Cross-correlation analysis, averaged across wells, exhibited a peak lag at −150 min. (Gray square and line: median and interquartile range of cross-correlation peak positions of the individual wells.) (*C*) Effect of IWP-2 treatment on the normalized power and normalized *T* signal compared to control (DMSO) samples (thin lines: individual well trajectories; bold lines: mean; *P*-values: permutation test of the treatment × time interaction for both normalized power and normalized *T* signal). Images represent examples for each treatment condition and do not share the same intensity scale. (Scale bar, 200 μm.) (*D*) Time courses of spatial mode fractions in DMSO and IWP-2 conditions following Fourier decomposition. Bold lines represent mean of n=8 replicates per condition and shaded regions are S.E.M.

Although the previous experiments identified a role for aggregation in *T* patterning, it remained unclear whether changes in *T* expression due to additional signaling-dependent effects occurred concurrently or instead followed a specific temporal order. As a readout of signaling state, we therefore quantified cell-level *T* expression during ePSM pattern formation. Over the course of the experiment, the distribution of *T* expression across the cell population broadened while the mean *T* level changed comparatively little. This indicates that some cells increased whereas others decreased *T* expression over time (Fig. 2*A*). This widening of the expression distribution became most apparent in the second half of the experiment, beginning around 500 to 600 min post dissociation.

We next compared these cell-level expression changes with the onset of spatial pattern formation by performing a cross-correlation analysis between the coefficient of variation (CV) of single-cell *T* expression and the normalized power of the emerging pattern. Relative to normalized power, the increase in *T* CV was delayed by several hundred minutes (Fig. 2*B* and *SI Appendix*, Fig. S5*A*), indicating that the first detectable evidence of pattern formation appeared before substantial divergence in cellular *T* expression. We then repeated the analysis using normalized power quantified from images of the constitutively expressed H2B-mCherry to compare expression changes more directly with density changes resulting from the aggregation-driven component of patterning (*SI Appendix*, Figs. S5*B* and S6*A*). Again, the increase in normalized power preceded the increase in *T* CV by several hundred minutes.

We additionally asked whether the delay in CV could be the result of a reporter-specific delay. We repeated a similar analysis using dissociated cells from an endogenous knock-in reporter line for *β*-catenin, a key WNT-signaling effector and upstream regulator of *T* expression (46). Nuclear *β*-catenin, indicative of active WNT signaling, changed its spatial pattern and cell-to-cell variation on timescales similar to those observed for the *T* reporter (*SI Appendix*, Fig. S6 *B*–*E*). Together with the strong correspondence between *T* reporter and *T* protein levels throughout patterning (*SI Appendix*, Fig. S2*C*), these results argue that the delayed increase in cell-to-cell *T* variation is not readily explained by an intrinsic delay in the *T* reporter.

Finally, we asked whether the late increase in *T* expression observed in a subset of cells is required for pattern formation by blocking WNT ligand secretion throughout the experiment with IWP-2 (47). IWP-2 reduced the mean *T* reporter signal but did not prevent the emergence of spatial patterning (Fig. 2*C*). However, patterns in IWP-2-treated wells appeared more fragmented than in controls (Fig. 2*C* and *SI Appendix*, Fig. S7 *A* and *B*). This disruption was corroborated by immunostaining for *T* protein, which further suggested that the correlation between cell density and *T* expression was weakened under these conditions (*SI Appendix*, Fig. S7 *C* and *D*). Consistent with this fragmented appearance, analysis of individual Fourier modes showed that IWP-2 flattened the late rise of the largest spatial modes associated with the characteristic *T* spot spacing (Fig. 2*D*). Together, these results suggest that WNT-signaling-dependent increases in *T* expression act to reinforce, rather than drive, the developing pattern.

Collectively, our dissection of *T* patterning dynamics supports a temporal sequence in which aggregation drives early spatial patterning, whereas subsequent WNT-signaling-dependent changes reinforce the developing *T* pattern. This temporal ordering is qualitatively consistent with recent observations in gastruloid models of A–P patterning (48, 49), indicating that similar dynamical ordering can arise across distinct in vitro models of A–P patterning.

### Quantifying the Biophysical Parameters of T-Expressing Cell Aggregation Using Nonlocal Continuum Modeling.

Given the central role of aggregation in *T* patterning, we sought to theoretically determine if this mechanism alone accounts for our observations. Aggregation-driven pattern formation requires that directed cell motion due to long-range cell–cell attraction overcomes undirected, random cell movements (20, 50, 51). To quantitatively characterize the random and directed motion of *T*-expressing cells in our system, we analyzed the early dynamics of *T* patterning using a nonlocal continuum theory (52–54). Motivated by the observed relationships between *T* reporter activity, local cell density, and aggregation-directed radial movement (Fig. 1*D* and *SI Appendix*, Fig. S4 *E* and *F*), we use a minimal model describing the dynamics of the high-*T*-expressing population, herein referred to as *T*+. The equation describing *ρ*, the density of *T*+ cells, reads

$$\begin{array}{c} \begin{array}{c} \partial_{t}\rho =\nabla \cdot (D\nabla \rho -\rho \left(1-\frac{\rho }{\rho_{\text{max}}}\right)[\chi \int \int_{\Omega }G\left(r^{\prime }-r\right)\rho \left(r^{\prime }\right)\text{d}r^{\prime }+B\left(r\right)]). \end{array} \end{array}$$

**Fig. 3.**
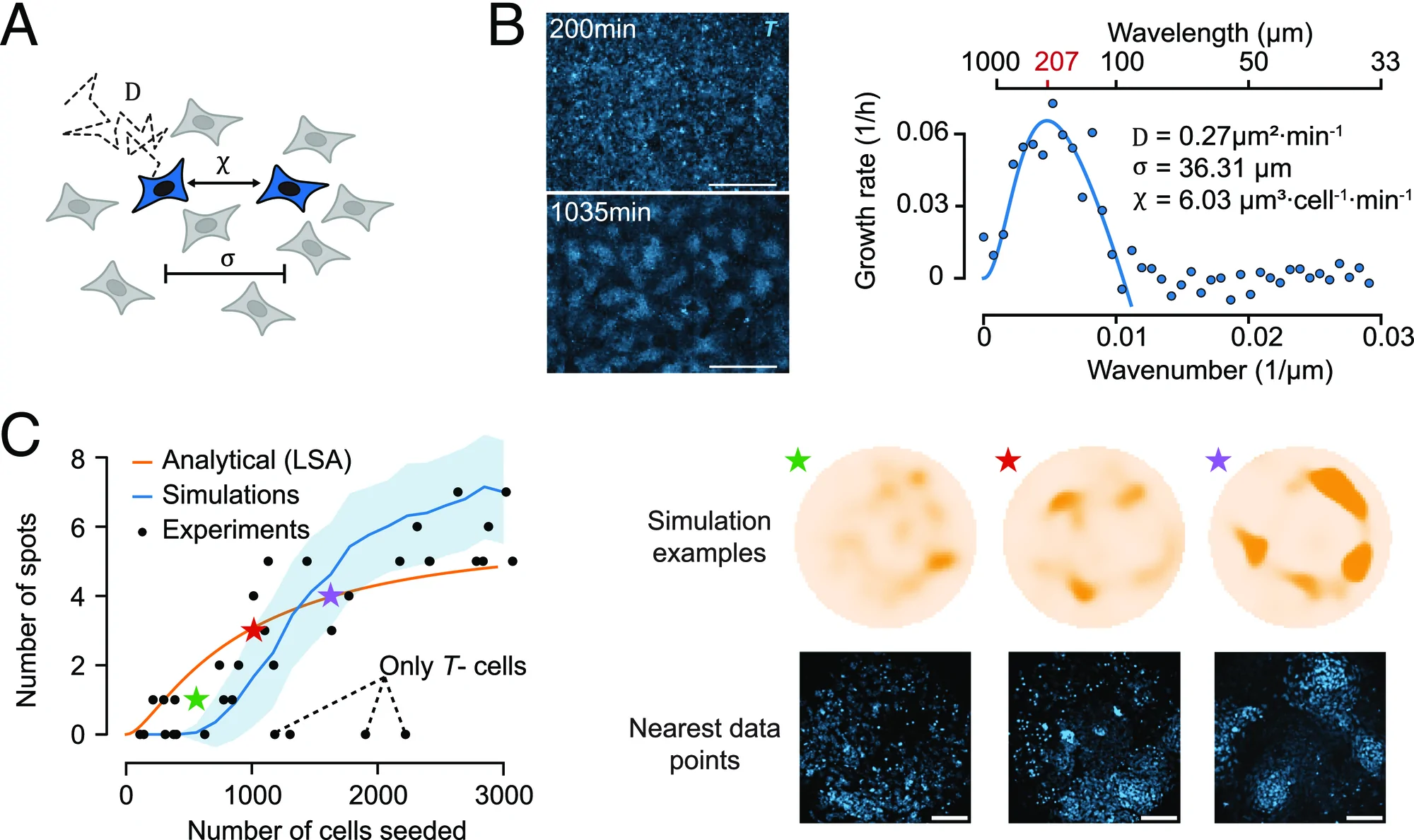
Cell movements determine *T* patterning dynamics. (*A*) The theory considers the distribution of *T*+ cells (blue), which move with a random component (effective diffusion coefficient *D*) and a directed component due to cell–cell attraction with strength *χ* and range *σ*. (*B*) Fitting the experimental dispersion relation from patterning experiments performed on 2 mm × 1.5 mm wells (*Left* panels) allows us to estimate the cell movement parameters (best-fit values for one out of three replicates shown). (Scale bar, 500 μm.) The wavelength indicated in red is the one with maximal growth rate according to the fitted curve. (*C*) Simulations on 500 μm wells with different initial densities show spot counts similar to experiments with varying cell seeding number and initial *T*+ cell fraction. Shaded area: SE of simulations. Stars mark selected example simulations, shown together with the closest experimental images. Three wells contained only anterior cell fates with low *T* activity. (Scale bar, 100 μm.) An analytical estimate based on the linear stability analysis provides an estimate for the number of spots to expect in the absence of nonlinear effects (orange line).

In this framework, an effective diffusion coefficient *D* sets the strength of random cell movements, and cell–cell attraction between *T*+ cells is described by a nonlocal term, parameterized by an interaction strength *χ*. The interaction kernel **G** describes how cell–cell interaction strength depends on distance. In this work, we use an exponentially decaying interaction with characteristic range *σ* (Fig. 3*A* and *SI Appendix*, *Supplementary Materials and Methods*). The term $1-\rho /\rho_{\text{max}}$ is called a packing term, which ensures that local velocity goes to zero when the density approaches $\rho_{\text{max}}$ and prevents aggregation to very high densities (55). The cells move within a confined space denoted by Ω, and the function **B** describes interaction effects with material outside of this space. Here, we use so-called neutral boundary effects, which assume the outside material has an attractive effect similar to that of the cells (*SI Appendix*, *Supplementary Theory*).

At the onset of patterning, when the density distribution is close to a spatially uniform profile, a linear stability analysis can be used to investigate the dynamics and typical length scales of emerging patterns. In particular, the spatial distribution of *T*+ cells (*SI Appendix*, Fig. S8) can be decomposed into spatial modes, each with a specific wavelength—similar to the analysis used to quantify patterns in Fig. 1*F*. The linear stability analysis allows one to calculate which of these modes grow or decay, and thus are expected to contribute to the pattern. Calculating the growth rate *ω* as a function of the mode’s wavenumber yields a dispersion relation valid for large systems (*SI Appendix*, *Supplementary Theory* and Fig. S9*A*)

[1] $$\begin{array}{c} \omega =-Dk^{2}+2\pi \chi \sigma \rho_{0}\left(1-\rho_{0}/\rho_{\text{max}}\right)\frac{k^{2}}{{\left(1+\sigma^{2}k^{2}\right)}^{3/2}}. \end{array}$$

which depends on the biophysical parameters of cell movement D,σ,χ and the maximum and average density $\rho_{\max}$ and $\rho_{0}$ respectively. In this equation, the wavenumber *k* is related to the mode’s wavelength *λ* by k=2π/λ.

To fit this theoretical relation to experimental measurements, we observed patterning dynamics in larger wells (2 mm × 1.5 mm) where boundary effects are negligible (Movie S4). Fourier analysis of the *T*+ cell density field over time showed that the spatial modes corresponding to patterning grew exponentially for a 500 min interval (*SI Appendix*, Fig. S9 *B*–*F*). This allowed a growth rate to be fitted to each mode and the experimental dispersion relation for each replicate well to be determined.

Fitting the theoretical curve to the experimentally obtained dispersion relation, we inferred the biophysical parameters underlying aggregation-driven patterning independently for each of the three wells (Fig. 3*B* and *SI Appendix*, Fig. S9 *G* and *H*). To arrive at a single set of parameters for subsequent analysis, we calculated the average and associated SD of the n=3 experiments. We obtained an average effective diffusion coefficient of *D* = 0.25 ± 0.07 μm^2^ min^−1^, an average cell–cell attraction strength of χ=5.8 ± 0.9 μm^3^/min/cells, and an average interaction range of σ=41 ± 8 μm. A complementary approximate Bayesian computation analysis showed that the experimental dispersion relations are able to constrain each parameter to a localized region of parameter space (*SI Appendix*, Fig. S10).

The magnitude of the random component, *D*, is similar to values obtained for cell movement in crowded environments for other multicellular systems (56–59). The units of $\chi \rho_{0}$ are those of a velocity, and the maximal value of the speed at a point in our model due to an attracting region of constant density *ρ* is 2χρ (*SI Appendix*, *Supplemental Theory* and Fig. S11 *A*–*C*). We thus expect $\chi \rho_{0}$ to give an order-of-magnitude estimate of typical cell velocities. Using the estimated $\rho_{0}$ from experiments and the fitted value of *χ*, the product of the two values is 0.04 μm min^−1^. This estimate of the velocity component due to cell–cell interactions is consistent with radial velocities obtained from single cell tracking (Fig. 1*E*). At the fitted interaction range, *σ*, the interaction strength has decayed to approximately 37% of its maximum at 4.8±1.0 nuclei spacings and 10% of its maximum at 11±2 nuclei spacings, indicating that cell–cell interactions extend beyond immediate neighbors (*SI Appendix*, Fig. S11 *D*–*F*). The fitted parameter values place the system well within the pattern-forming regime predicted by the theory (*SI Appendix*, Fig. S12*A*). The fastest growing modes of the fitted dispersion relations correspond to pattern wavelengths of 220 ± 30 μm, differing from the mean value of the nearest-neighbor distance between *T* spots by 1.8 SDs (*SI Appendix*, Figs. S3*C* and S9 *F*–*H*). A scaling analysis shows that the initial pattern’s wavelength *λ* and growth rate *ω* scale, to leading order, as

[2] $$\begin{array}{c} \lambda \propto \sigma \;\omega \propto \frac{\chi }{\sigma }\rho_{0}\left(1-\rho_{0}/\rho_{\max}\right). \end{array}$$

The wavelength is thus proportional to the interaction length *σ*, and the growth rate is inversely proportional to *σ* (*SI Appendix*, Fig. S12 *B* and *C* and *Supplemental Theory*). This result, applicable to large systems, agrees with the intuition that to establish large-wavelength patterns, cells need to move across larger distances, which takes more time.

To validate our parameter estimates, we performed numerical simulations of the continuum equation using the fitted values without additional free parameters for different initial cell densities, and compared the results to experiments in which we varied the *T*+ fraction and total cell seeding number (*SI Appendix*, Fig. S13 *A*–*C*). We obtained a close quantitative agreement between simulations and data, confirming the predictive power of the inferred parameters (Fig. 3*C*). Moreover, these results qualitatively agree with the analytically obtained result that increasing the density makes the wavelength of the pattern smaller, which means more spots on a given system size (*SI Appendix*, Fig. S12*D* and *Supplemental Theory*). The apparent difference between the analytical and simulated estimates at low densities can be explained by the small dominant growth rates in this regime, which entail that the predicted spots may only emerge at times longer than those simulated.

In summary, both analytical and numerical results support our experimental findings that aggregation-driven patterning underlies the initial distribution dynamics of *T*+ cells. Our estimates of motility and interaction parameters show that this system is robustly capable of spontaneously forming density patterns.

### Aggregation Dynamics Couple System Size to Patterning Timescales.

**Fig. 4.**
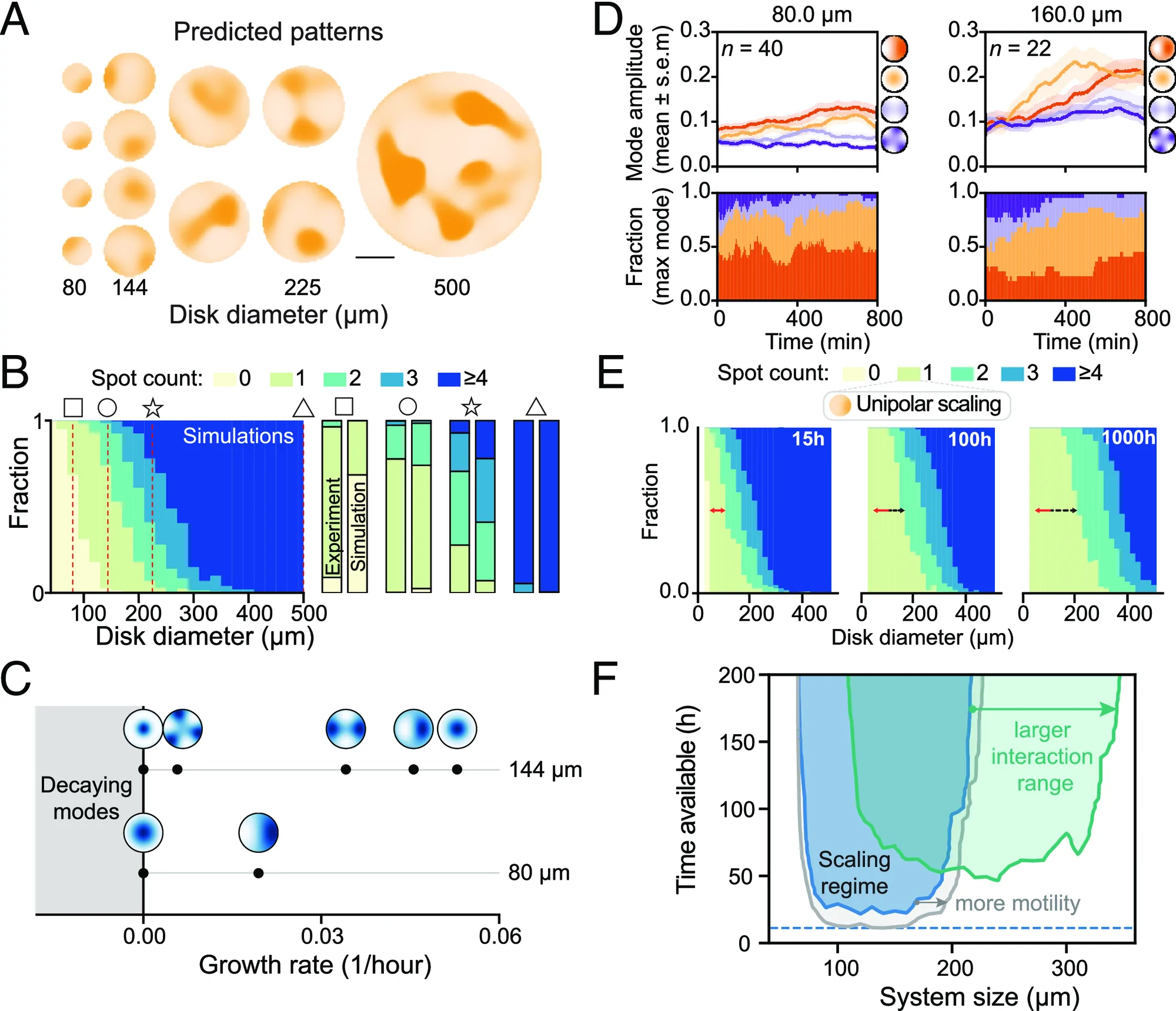
Finite-size and nonlinear effects explain patterning outcomes across system sizes. (*A*) Representative simulations show patterning outcomes for disks of different sizes at t=1,100 min. (Scale bar, 100 μm.) (*B*) The distribution of spot numbers across simulations changes as a function of disk size. For the experimentally realized disk sizes (red dashed lines and icons), we compare spot count statistics between simulations and data shown in Fig. 1*B*. (*C*) Growth rates and shape of spatial modes with zero or positive growth rates obtained from a linear stability analysis on disks. (*D*) We project the time-lapse disk data onto four selected modes and compute their amplitude (*Top*, cross-disk average) and the distribution of the dominating modes (*Bottom*) over time. Shaded area is S.E.M. (*E*) Spot count statistics for simulations conducted past typical experimental durations show how the scaling region of the unipolar pattern increases (black dashed arrow, with red arrow referencing the scaling width at 15 h). (*F*) Performing simulations across time windows and system sizes (100 runs per point), we compute the unipolar scaling regime for the experimental ePSM parameters (blue region), for a two-fold increase in cell motility D and *χ* (gray region), and a two-fold increase in the interaction range *σ* (green region). We define the scaling region as the set of points for which at least 50% of simulations exhibit a single spot.

While quantifying the patterning dynamics in large wells allowed us to characterize the pattern-forming mechanism in the absence of boundary effects, the size and shape of the space within which patterning takes place can strongly influence its outcomes (60). The effects of system size, shape, growth, and deformations have been extensively studied for (mechano)-chemical patterning systems (61–65). For example, a generic effect of size is that, in confined spaces, only a discrete set of pattern modes whose wavelengths fit within the available space can develop (66, Chapter 2]. With estimates of all model parameters in hand, we used simulations to investigate pattern formation on small disks, and generated fit-free predictions of patterning outcomes as a function of system size to compare with the results of Fig. 1*B*. We found multispot patterns on larger disks, and unipolar patterns on a range of smaller disks (Fig. 4*A*). Using initial conditions based on experimental density profiles at 200 min, the independently parameterized simulations showed good agreement with experiments in the spot-count distributions for 144, 225, and 500 μm disks with the main discrepancy occurring for the 80 μm disks (Fig. 4*B*). We verified that, among unipolar outcomes, spot size increased linearly with system size in simulations and was consistent with experiments (*SI Appendix*, Fig. S14). We then defined the unipolar scaling regime as the range of disk sizes for which 50% or more of the patterns were unipolar. Under this criterion, experiments showed a scaling regime from 80 to 144 μm, while in our simulations the scaling regime was 100 to 160 μm.

To identify the mechanisms underlying this scaling behavior, we performed a linear stability analysis adapted to circular domains (*SI Appendix*, *Supplementary Materials and Methods*). In contrast to infinite domains, where Fourier modes are appropriate, in this case the modes change with domain size and parameter values. Our analysis showed that initial patterning dynamics can be described by only a few growing spatial modes (Fig. 4*C*). For 80 μm disks, only a single spatial mode can grow: the unipolar pattern. For 144 μm disks however, single-spot and two-spot patterns have similar growth rates, indicating that initially multiple spots may form. The size of the system thus strongly constrains the possible patterns.

To investigate whether the temporal dynamics of the experimental system can be described by these unstable modes, we collected a dataset of time-resolved *T* patterning dynamics on small disks. After filtering low-density disks and disks with cells that enter at later time points, we decomposed the remaining observed density patterns into spatial modes. We used the modes obtained by the linear analysis and analyzed the contributions of four modes: central spot, unipolar, bipolar, and tripolar (Fig. 4 *C* and *D* and *SI Appendix*, Fig. S15 *A* and *B* and Movie S5). These results confirm a number of predictions from the linear stability analysis. First, for 80 μm diameter disks, the polar mode is dominant and growing, but its growth rate is smaller than the dominant growing modes on the larger disks. Additionally, for 160 μm diameter disks, both central-spot and polar modes have large growth rates, whereas two- and three-spot modes grow at a slower rate, also consistent with the linear analysis.

At later times, the polar mode becomes dominant on the 160 μm disks and the other modes drop in amplitude, suggestive of nonlinear effects. Indeed, on some 160 μm disks a cell cluster that formed in the center drifted to the side at later times (second example in *SI Appendix*, Fig. S15*B*). This observation is consistent with our previous theoretical investigations of one-dimensional aggregating systems that show the same behavior (54).

In larger systems imaged over longer periods, we also directly observed the joining of initially separate *T*+ spots (Movie S6). Such merger events are characteristic of coarsening, a nonlinear phenomenon, in aggregation-driven systems (67, 68). Coarsening can in principle enable the scaling of a unipolar pattern, i.e. leading to a single *T*+ domain, across arbitrarily large systems. However, the time required to reach this state increases nonlinearly with system size because the intermediate multispot patterns are long-lived transients in the system dynamics (54).

To investigate quantitatively how patterning duration influences this scaling capacity for aggregating multicellular systems, we investigated spot-count statistics as a function of system size in long-term simulations of up to 1,000 h. For such long simulations, we observed an approximately 2.5-fold increase in the width of the unipolar scaling regime, confirming that the time available for a given patterning process effectively acts to limit the size range over which patterns can scale (Fig. 4*E*). Conducting simulations over a wide range of system sizes and developmentally relevant time windows, we mapped out the full unipolar scaling regime for the aggregation parameters of the experimental system (Fig. 4*F*, blue region), and found that predominantly two effects determine the shape of the scaling regime. First, the patterning timescale imposed by the parameters D,σ and *χ* (horizontal dotted line) sets a lower bound for patterning time. Second, the finite size effects and nonlinear coarsening effects shape the boundaries of the scaling regime on longer timescales. To see how the scaling regime depends on the biophysical parameters controlling aggregation dynamics, we simulated the effect of a two-fold increase in the motility parameters *χ* and *D*, and the interaction range *σ*. We found that increasing motility expanded the scaling regime modestly in all directions, mainly due to the global speedup of all cell motion, which mathematically corresponds to a rescaling of time. A larger interaction range, on the other hand, increased the time required to develop a pattern while shifting and expanding the size range over which unipolar patterns dominate due to a combination of finite-size and long-term nonlinear effects. Since the dominant growth rate is inversely proportional to *σ*, it is indeed expected that systems with larger interaction ranges develop patterns more slowly (*SI Appendix*, Fig. S12*C*).

Together, our results demonstrate how aggregation dynamics links patterning time scales to system size and delineates mechanisms for controlling the scaling of unipolar patterns.

## Discussion

Primary cells derived from the PSM retain a robust ability to self-organize into structures characterized by multipolar *T*-positive domains, a pattern that departs from the unipolar *T* domain observed in situ (Fig. 1*B*, 35). We harnessed this potential to address how cell fate patterns depend on system size and identified a regime in which unipolar *T* patterns reliably form and scale to overall size. Experiments and theory revealed a key principle setting the size–patterning relationship in aggregating systems: scaling is robust for small system sizes, but for larger systems the time required to achieve scaling exceeds realistic developmental timescales. The underlying attractive cell–cell interactions therefore impose a time–size constraint on pattern scaling, setting an upper system size that can achieve a unipolar outcome within a limited patterning window. Moreover, because the theory depends on an effective interaction rather than on a specific mechanism, the resulting constraint applies to any mechanism that produces comparable aggregation dynamics.

Importantly, this scaling limit is not fixed but reflects a fundamental time–size tradeoff. For a given developmental time window, only a restricted range of system sizes successfully achieves unipolar scaling. Both linear and nonlinear effects arising from aggregation-driven patterning shape this tradeoff: finite-size effects limit the possible number of initially forming aggregates, delineating a range of system sizes in which unipolar patterns are the only ones that can form. Beyond this range, larger systems support initial patterns with multiple aggregates that subsequently undergo slow merging toward a final unipolar state—an outcome that may not be reached within the available time window. Our results therefore suggest that system size must be constrained in accordance with the time available to achieve unipolar outcomes.

The size range over which patterns scale can be enlarged by modifying the effective parameters governing cell movement and attraction. Our simulations show that increasing motility primarily accelerates the dynamics, whereas changing the interaction range alters both the characteristic spatial scale and the time required for pattern formation. These parameters could arise from different molecular or cellular mechanisms. In some cases, more detailed descriptions can be mathematically recast as nonlocal advection equations such as the one used here (54, 69). The Keller–Segel model, originally motivated by chemotaxis-driven aggregation in *Dictyostelium*, provides one such example (50) while alternative interaction rules can be represented by different interaction kernels (70). The value of the interaction range *σ* we inferred extends over several cell diameters or neighbor distances and is compatible with longer-range processes such as traveling chemical waves (27, 28), cellular protrusions (21, 71), or stress propagation through the tissue (23, 25). At the same time, local adhesion-mediated sorting may contribute alongside longer-range interactions, as reported in recent studies (22, 48, 49). Perturbing these candidate mechanisms and measuring their effects on random motion, attraction strength, and interaction range would reveal how molecular and cellular regulation shifts the scaling regime predicted by the model.

Whether embryos face comparable time–size constraints remains unknown, but early mouse gastrulation provides a plausible context in which to investigate this possibility in situ. Between E6.0 and E6.5, the embryonic portion of the epiblast has linear dimensions of approximately 150 to 200, close to the boundary of the scaling regime identified here (72). Across these early developmental stages, Rac1-dependent migration contributes to polarization of the anterior visceral endoderm and directional redistribution of nascent mesoderm, while reported mesodermal cell velocities of 0.1 to 0.3 and approximately 0.7 μmmin^−1^ are similar in magnitude to those measured in our ex vivo system (73–75). These similarities in spatial scale and movement rate motivate the hypothesis that movement-driven patterning in the embryo may encounter related time–size constraints.

Within complex tissues such as the developing embryo, aggregation-driven patterning occurs concurrently with cell signaling, proliferation, and global shape changes. How these factors influence pattern scaling depends on the relative timescales between these processes. In the simplified context we studied, chemical inhibitor experiments showed that WNT-dependent regulation altered both *T* expression and spatial patterning, demonstrating that cell-state regulation can modify aggregation-driven patterns. A direct extension of the current model could therefore allow the average *T*+ density to change through transitions between high- and low-*T* states, while addition of a cell-turnover term could additionally account for changes in population size. An alternative approach would be to couple spatial patterning to an intracellular gene-regulatory network as was recently done within a reaction–diffusion framework (76). Such extensions would test how regulation of the *T*+ population modifies the aggregation-driven time–size tradeoff, including whether changes in cell-state composition stabilize multipolar outcomes rather than permitting eventual convergence to a single domain (77). More broadly, the strong geometric constraints identified in this work raise the question of how living systems use size and shape to steer self-organized patterns. Embryo geometry influences how gastrulation proceeds during development (78), while confinement of organoid cultures is recognized as an important factor for improving reproducibility (79). These examples provide empirical evidence for a guiding role of geometrical constraints and suggest future avenues for harnessing the principles identified here.

## Materials and Methods

Mice used in this study were housed under veterinary supervision and managed by Laboratory Animal Resources at EMBL. All animal experiments were approved by the EMBL Institutional Animal Care and Use Committee (project codes: 21–001_HD_AA and 25-014_HD_AA). Briefly, primary cells from the tailbud and posterior presomitic mesoderm from E9.5–E10.5 mouse embryos were mechanically dissociated from the tissue and cultured on fibronectin-coated substrates of defined size. Fixed cell and live cell fluorescence images were acquired on laser scanning confocal microscopes. A custom trained StarDist3D model was used for nuclear segmentation while Ilastik was used for cell tracking. Particle image velocimetry, spatial correlation analysis, and Fourier-based analyses were used to quantify cell movement and pattern formation in MATLAB. Aggregation dynamics were described with a nonlocal advection–diffusion model whose parameters were inferred from experimentally measured dispersion relations and used without further fitting to predict patterning outcomes across system sizes and densities. Complete description of animal procedures, cell isolation and culture, micropatterning, imaging, image analysis, statistical analysis, mathematical modeling, numerical simulations, and theoretical derivations is provided in *SI Appendix*, *Supplemental Materials and Methods* and *Supplemental Theory*.

## Supplementary Material

Appendix 01 (PDF)

Movie S1. **Dynamics of *T* patterning.** Time-lapse video of pattern formation in the *T* reporter channel beginning 200 minutes post tissue dissociation. Example z-slice is 5/13. Images captured at 5-minute intervals and scale bar = 100 μm.

Movie S2. **Before JLY washout.** Composite time-lapse video of *T* reporter (blue) overlaid on top of H2B-mCherry (gray) to show arrest of cell movement. Imaging begins 51 minutes after tissue dissociation and the addition of JLY. Washout is performed immediately after the last imaged frame. Video corresponds to the example in Fig. 1H from z-slice 3/13. Images captured at 5-minute intervals and scale bar = 100 μm.

Movie S3. **After JLY washout.** Composite time-lapse video of *T* reporter (blue) overlaid on top of H2B-mCherry (gray) to show recovery of cell movement. Imaging begins shortly after washout of JLY and ends 1,000 minutes after tissue dissociation. Video corresponds to the example in Fig. 1H from z-slice 3/13. Images captured at 5-minute intervals and scale bar = 100 μm.

Movie S4. **Dynamics of *T* patterning in large wells.** Top half of the video depicts pattern formation in the *T* reporter channel in large wells. In this example, cells are pooled together from 8 embryos. Bottom half corresponds to the *T*+cell density field after processing. Images tiled in a non-overlapping manner, captured at 5-minute intervals and scale bar = 100 μm.

Movie S5. **Dynamics of *T* patterning on small disks.** Composite time-lapse video of T reporter (blue) overlaid on top of H2B-mCherry (gray) to show examples of cell confinement on micropatterned disk substrates. In the video, three examples of 80 μm disks are shown in the top row and three examples of 160 μm disks are shown in the bottom row. Videos were captured at 5-minute intervals and scale bar = 50 μm.

Movie S6. **Dynamics of *T* patterning at longer times.** Time-lapse videos of pattern formation in the T reporter channel beginning 650 minutes post tissue dissociation and extending to 1,250 minutes. The left example is an extended version of Movie S1 while the right example is a separate example from another experimental day. White arrows are added annotations marking merging events. Example z-slice is 5/13. Images captured at 5-minute intervals and scale bar = 100 μm.

## Data Availability

Code and example data are available on GitHub (80). All raw imaging data used in this study have been deposited in the BioImage Archive under accession S-BIAD3927 (81). Simulation data, processed segmentation data, and cell tracks have been deposited in BioStudies under accession S-BSST3378 (82). All other data are included in the manuscript and/or supporting information.
